# EHMTI-0199. CGRP modulates trigeminal ganglionic neuronal excitability in minimally low pH condition

**DOI:** 10.1186/1129-2377-15-S1-F24

**Published:** 2014-09-18

**Authors:** U Jansri, S Bongsebandhu-Phubhakdi, A Srikiatkhachorn

**Affiliations:** 1 Faculty of Medicine Chulalongkorn University, Department of Physiology, Bangkok, Thailand

## Introduction

It is well recognised that trigeminal ganglionic neurons are responsive to acidic condition (pH<6.5). Their response to minimally low pH (7.0) and the interaction with calcitonin gene related peptide (CGRP) is not well demonstrated.

## Aims

To investigate the effect of minimally low pH (7.0) on trigeminal neuronal excitability and the modulating effect of CGRP upon this process.

## Methods

Whole cell patch clamp recording was performed in primary cultured rat trigeminal ganglionic neurons. Cultured neurons are classified to small-to-medium sized (diameter < 40 µm) and large neurons (diameter > 40 µm). Depolarizing current pulses were applied to generate action potential. Electrical properties were measured at pH 7.4 and re-measured again after incubation fluid pH was adjusted to pH 7.0. In another set of experiment, cultured neurons were pre-incubated with 1 µM CGRP for one hour prior to electrical recording.

## Results

In small-to-medium-sized neurons, lowering pH to 7.0 increased excitability of trigeminal neurons as evident from less negative resting potential, more negative threshold potential and decreased rheobase. Minimally low pH did not alter the excitability of large-sized neurons. After pre-incubation with CGRP, large-sized neurons became more sensitive to acid condition. The rheobase was decreased from 60.8 ± 9.2 to 51.7±8.5 pA (pH 7.4 and 7.0 respectively).

## Conclusions

These findings indicate that pH 7.0 can increase the excitability of small-to-medium-sized neurons. On the other hand, large-sized neurons will become more sensitive to acidic condition only after pre-exposure to CGRP. These results reflect the interaction between CGRP and low pH condition in modulating the excitability of trigeminal neurons.

No conflict of interest.

